# Incidence of educational mismatch and earning in Pakistan

**DOI:** 10.1371/journal.pone.0268008

**Published:** 2022-06-03

**Authors:** Muhammad Zaheer Khan, Rusmawati Said, Nur Syazwani Mazlan, Norashidah Mohamed Nor

**Affiliations:** School of Business and Economics, University Putra, Serdang, Malaysia; University of Milano–Bicocca: Universita degli Studi di Milano-Bicocca, ITALY

## Abstract

This study empirically examines the incidence and earning effect of educational mismatch in the labor market of Pakistan. Microdata obtained from the labor force survey for the years (2013–14, 2014–15, 2017–18) is used for the analysis. The realized match approach is used to measure the required level of education, and the augmented mincerian model is used to determine the potential earning effects associated with the educational mismatch. The study found a considerable incidence of under-education and over-education in the labor market. Results show a positive return to under-education and over-education. However, the return to the required level of education is significantly higher than both the under-education and over-education.

## 1. Introduction

The educational mismatch can have certain negative ramifications for individuals, such as job dissatisfaction, skill depreciation due to lower access to further education and training activities, lower labor productivity, underutilization, as well as wage penalty [1–4]. It can lead to low productive economic sectors and even jobless growth. The structural mismatch in the labor market could also cause unemployment and hinder investment and employment growth.

Developing countries (such as Pakistan) have specific labor market characteristics that might influence educational mismatch on income. Pakistan belongs in the lower-middle-income country group (World Bank classification) with an emerging market and developing economy (IMF). The analysis of educational mismatch and its impact on earnings in the labor market of Pakistan can be interesting for several reasons.

First, the country’s higher education sector has observed significant structural changes in the last two decades. Recent statistics show that over 209 public and private sector higher education institutes are producing around 500,000 university graduates each year. However, with the expansion in higher education, there appears to be the paradox of graduate unemployment. The educated unemployment (higher education and diploma) has increased significantly from 6.52% in 2003–2004 to 18.82 in 2017–2018. Second, with an average labor productivity growth rate of about 1.5%, Pakistan is positioned at the bottom of the lowest labor productivity rate in the region; while countries such as India, Sri Lanka, Bhutan, Vietnam, and Bangladesh have all witnessed strong labor productivity growth during the same period, even though the average annual hours of work by persons employed in Pakistan are almost equal to the average annual hours of work in India, Bangladesh, and Sri Lanka.

There is a general agreement that job-education mismatch would have a detrimental impact on productivity. Evidence suggests that employing workers in a position that requires less educational qualification than what the workers actually possess is potentially counterproductive [1], as it may lead to underutilization of skills and productivity decline [5]. Similarly, having individuals engaged in an occupation that requires higher than their actual qualification would also lead to low productivity. There is a strong possibility that one of the reasons Pakistan faces low productivity could be a skill imbalance in the labor market because employee skill sets are mismatched to the demand of a particular job.

Apart from that, young people have a significantly strong desire to leave the country. World Bank’s statistics from the Migration and Remittances Factbook 2017 show that Pakistan stood third in South Asia (after India and Bangladesh) and sixth globally (after India, Brazil, Russia, China, and Bangladesh) for human capital mobility. The statistics from the Pakistan Bureau of Emigration and Overseas Employment and the Pakistan Economic Survey (various issues) show that Pakistan’s stock of immigration rose (those seeking employment abroad) from one million in 1981 to 8.77 million by December 2017. This scenario is unlike the countries such as New Zealand, where the departing highly skilled labor is being substituted by highly qualified individuals from many other countries; [6] referred to this phenomenon as "brain exchange" rather than "brain drain”.

A very few studies related to educational mismatch can also be found in Pakistan [7–9]. However, the existing literature lacks the sense that they do not provide a detailed description and analysis of the incidence of educational mismatch in the labor market. This study aims to contribute to the existing literature of developing countries, especially Pakistan, in several ways. First, using the pooled microdata for the period (2013–14, 14–15, and 17–18), this study provides a detailed description of educational mismatch in the labor market by gender, level of experience, and nine major occupations based on international occupational classifications across industries. Second, this study investigates the impact of educational mismatch on earnings by gender and across occupations. It also investigates whether the effects of educational mismatch differ for males and females in different age groups. It is noteworthy that due to data limitations, this study only deals with the vertical educational mismatch.

The rest of the paper is organized as follows: Section 2 contains a brief review of theoretical and empirical literature related to educational mismatch and its impact on earnings, followed by a detailed description of data and methodology in section 3. Section 4 presents the findings of this study, followed by the conclusion and recommendations in section 5.

## 2. Literature review

The neo-classical model of human capital theory [10] suggests that every worker’s average efficiency is paid according to the level of human capital he has accumulated. The model, therefore, allows no mismatches. However, since wage premium depends upon human capital, other factors, such as years of experience, can offset the qualification mismatch. If someone has been paid a lower than the corresponding market rate to his educational qualification, either the level of education does not fully accumulate the individual’s level of skills or is, as in [11], a temporary phenomenon where people first accept lower-paying jobs if the job offers a greater likelihood of advancement.

The signaling theory [12] states that education is used to identify more productive, capable, and ambitious workers in an imperfect labor market. As a result, individuals invest more in education in the hope of establishing a competitive advantage over other job applicants. The job competition model [13] views surplus education as a demand-driven, long-term labor market problem, implying that job characteristics rather than personal characteristics determine productivity and earnings. Workers compete for jobs based on their training costs in the job competition model. Employers prefer over-educated workers because it saves training costs. In this way, over-education serves as an alternate for training.

The Career mobility theory [11] can be considered an extended version of human capital theory. It states that individuals with high education levels may accept a job for which they are over-educated, enabling them to gain specific human capital characteristics, i.e., job-specific experience and training. It allows them to accumulate skills that can be used later to switch to higher-level jobs.

Assignment theory [14] holds that job characteristics and individual human capital determine labor’s marginal product (the wage rate). Workers’ characteristics differ in a dynamic economy, as do the job complexities; assigning differentiated jobs to heterogeneous workers creates the allocation problem. As a result, the educational mismatch will continue to be a persistent feature of the labor market.

The empirical literature on the impact of educational mismatch and earnings has emerged with the seminal work of [15], who proposed an extended or augmented version of the basic mincerian model. The model decomposes the attained level of education into over-education, required-education, and under-education. They found a significantly smaller return of over-education than the required level of education. Subsequently, several studies confirm their findings, for example, [16–20] and [5]. Some Studies have also accounted for worker heterogeneity in measuring the educational mismatch and earning differentials [21–23].

It has been well established in the literature that every occupation requires a certain level of education [24, 25]. Over-education is a scenario in which a worker requires less qualification than he has attained to be employed and perform in a specific occupation. Some studies have shown positive results of over-education in professions that require lower qualifications. However, employers value job specific human capital by offering higher wages to workers who are the best fit for a particular job. If an individual’s human capital is not at par with the job specific human capital, in this case, the workers are considered less productive. Employers do not pay a high premium for human capital that does not contribute to labor productivity, and over-educated workers would earn less than the workers with a qualification that is well matched for that specific job [2, 19–21, 26].

Similarly, individuals with a lower than required level of education for occupation are classified as undereducated. Several empirical studies on the incidence of educational mismatch found heterogeneous results for developing countries [27, 28] found evidence of a high ratio of under-education for countries such as Cambodia, Jordan, Liberia. More recently [29], found a higher degree of under-education in Vietnam than over-education. The study states that the reason for significant under-education in Vietnam is because a considerable proportion of the population lives in rural areas with limited access to formal education facilities. The study also stated no significant penalties or gains associated with being under or over-educated in the labor market. Some other studies have found evidence of the negative impact of educational mismatch on workers’ income in developed countries [29–34].

In analyzing the Egyptian labor market, [32] also found significant evidence of over-education. However, the study revealed a tradeoff between over-education and years of experience. The negative effect of being over-educated in the labor market declines with years of experience. Similarly [35], also found a negative correlation between education quality and over-education in the EU-15. In another recent study [36] found evidence of a significant incidence of over-education in Bosnia-Herzegovina and low return to over-education compared to those with the matched level of education. Analyzing the educational mismatch and return to education in the Brazilian labor market [37] identifies the over (under) education with an almost 50:50 ratio in the labor market. The results also revealed a significant wage penalty of being over-educated (undereducated) in the labor market, with mismatched workers earning a significantly higher (lower) than those with a well-matched educational level.

In the case of Pakistan, some researchers have focused on the issue of education mismatch and its impact on earnings in Pakistan; however, most of the previous studies are very descriptive in nature and limited in scope; see, for example, [38–40]. In his pioneering work in the context of Pakistan, [7] used a primary dataset based on a survey of employed graduates (SEG) conducted in early 2010 in two cities of Pakistan (Rawalpindi and Islamabad) to provide evidence of educational mismatch among graduates in Pakistan. Results showed relatively higher undereducation compared to overeducation in two cities; however, the dataset doesn’t consider the gender difference. For the comparison purpose only, in the same study, the author used secondary data from the nationally representative Labor force survey (LFS) of Pakistan to show a relatively higher incidence of undereducation than overeducation. Overeducation was found to be high among females as compared to male workers. However, the study doesn’t provide further information about the educational mismatch across occupational distributions or the impact on earnings. [8] used the SEG, 2010 dataset to identify that over-qualified graduates face higher wage penalties in Rawalpindi and Islamabad. In a recent study, [9] used the microdata from the nationally representative household integrated survey of Pakistan (2013–14) and quantile regression to determine the impact of overeducation on wage distribution in Pakistan. The cross-sectional analysis indicates higher Earning differences among over-educated male workers. However, the results show no statistically significant earning difference across the distribution for over-educated female workers. Even though the study considers the impact of educational mismatch on earning distributions by gender, the study doesn’t provide details whether the distribution of incidence of educational mismatch across gender or occupations.

## 3. Methodology

### 3.1. Measurement of educational mismatch

There is a large body of literature on the gap between the actual level of education attained by workers and the job’s required level of education. Using the worker’s current job and level of attained education, these studies provide three measurements defined as years of over-education, required-education, and under-education. However, existing economic literature shows several methods for measuring these variables. These measurement methods can be categorized into two major groups: subjective and objective methods. The former includes (1) indirect self-assessment, according to which the workers are asked about the required education for a specific job. Then the worker’s attained level of education is compared with the job’s required level of education. (2) direst self-assessment; according to which the workers are asked directly whether they are over or undereducated for their current jobs.

The educational mismatch can also be assessed based on the objective method: (1) using the Job Analysis (JA) approach. Professional job analysts compute the academic requirements for specific occupations by grading each occupation to determine an individual’s required education and comparing it with their actual education level. (2) by realized match approach, which can be classified into two categories. The first is by calculating the mean and standard deviation of the education level of individuals as the required level of education for a specific group of occupations [41]. The individual is considered mismatched if his education level is greater (less) than the mean education plus (minus) one standard deviation for that specific group of occupation. While the second method is based on obtaining the mode value rather than the mean to determine the required level of education in a particular occupation [42]. The individual is considered mismatched if his education level falls above (below) the mode of education level for that specific occupation.

Because of the data limitations, it is not possible to use the subjective methods method in this study. Therefore, this study is based on realized matched approach, and following [18, 28, 42], the mode of attained educational qualification is used as the required level of education across occupations for further analysis.

### 3.2. Data description

This study is based on three waves of the Pakistan labor force survey for the years 2013–2014, 2014–2015, and 2017–2018). The labor force survey conducted by the statistical bureau of Pakistan is a cross-sectional survey that provides microdata incorporating annual estimates of detailed labor market characteristics. The survey is being conducted either annually or every second year. The sample for this study includes the civilian labor force working-aged between 15–65 working full time in different sectors of the economy. The sample is limited to regularly paid employees with at least 35 weekly work hours. Own account workers and those with less than 35 weekly hours of paid work are excluded from the sample. A complete list of variables is given in Table A1 in S1 Appendix.

Mapping of education qualification of Pakistan based on the International Standard Classification of Education (ISCED) is used to account for the actual level of education obtained by the individuals. Further, occupational categories (excluding Armed forces) based on ISCO-08 have been used to classify the level of required education (RE), over-education (OE), and under-education (UE) for each worker working in a specific occupational group.

Given the specific nature of the labor force survey, the data are pooled together for analysis. The required level of education (re) is estimated at occupation level, and the variables are pooled across years by gender.

### 3.3. Empirical model

There are mainly two approaches used to determine the impact of educational mismatch on income. While both methods use a modified mincer earning function for analysis, the first approach uses the dummy variables to represent over-education and under-education. The second approach based on [15] breaks the actual years of education into over-education, required-education, and under-education (ORU specification) as follows:

AE={RE+OE}–UE (1)

Where AE is the level of attained education, RE is the required level of education for a specific occupation, OE is the amount of education acquired by an individual that exceeds the required level of education in a particular occupation, and UE is the amount of education attained by an individual that is less than the required level of education in an occupation. OE will be zero for undereducated and correctly matched individuals. UE is zero for over-educated and correctly matched individuals. To calculate the level of over-education and under-education, the Eq (1) can be reduced as:

OE={AE–RE}ifAE>RE,0otherwise (2)

UE={RE–AE}ifAE<RE,0otherwise (3)

After inserting the over-education, under-education, and required level of education in mincer wage equation, the augmented equation takes the following form:

$$lnW_{i}=\beta_{0}+\beta_{1}{OE}_{i}+\beta_{2}{RE}_{i}+\beta_{3}{UE}_{i}+\beta_{4}{exp}_{i}+\beta_{5}{exp}_{i}^{2}+\beta_{6}X_{i}+\mu_{i}$$ (4)

In Eq (4), the log monthly wage of a worker is denoted by W. X is the vector of all other control variables, including marital status, the number of children at home, area of residence (urban or rural), province, occupation, industry and years dummy.

## 4. Results and discussion

Table 1 presents the summary statistics of key variables used in this study. After excluding observations with missing values, the total sample of this study comprises 64,964 individuals, among whom only 14.15% are female. Table 1 shows the difference in the log of monthly earnings of males and females; on average, males earn higher than females. Statistics on education level based on ISCED classifications show a significantly higher percentage of females in tertiary education than males. Table 1 also presents the average years of required and obtained years of schooling. The required level of education is higher for females as compared to males. The low level of participation can be due to two reasons: first; traditional gender roles assigned to females in the society of Pakistan, and even though there has been a significant increase number of females obtaining a higher level of education; the total labor force participation of females in Pakistan is still the lowest in the region. Second, a significantly large proportion of the population lives in rural areas characterized by a large share of informal employment. Statistics on occupational distribution by gender based on ISCO classifications show that most females, working as full-time workers, cluster around highly skilled jobs. It also indicates the presence of occupational gender segregation in the labor market.

**Table 1:** Summary statistics.

	Total	Male	Female
Log m_I	9.07	9.13	8.57
Obtained education	11.27	11.01	13.45
Required education	11.94	11.82	15.24
Female %	14.15	-	-
Experience	18.68	19.36	17.15
Exper. Squared	535.3116	490.06	276.44
Married	74.44	76.48	57.46
Percentage of the labor force by Area of Residence			
Urban	52.17	51	53
Rural	47.83	49	47
Percentage of the labor force by level education			
Primary education	14.29	15.52	3.97
middle	13.41	14.51	4.20
Secondary	24.06	24.79	17.99
Higher Secondary	16.15	15.95	17.84
Graduates	31.62	28.81	55.17
Post Graduates	0.47	0.42	0.82
Total	100.00	100.00	100.00
Employment Percentage by Occupation			
Manager	7.00	7.44	3.32
Professionals	17.06	14.15	41.44
Technicians and associate professionals	20.75	17.79	45.52
Clerical support workers	9.10	9.97	1.80
Service and sales workers	17.03	18.84	1.96
Skilled agricultural, forestry, and fishery workers	0.86	0.95	0.07
Craft and related trades workers	8.75	9.66	1.16
Plant and machine operators and assemblers	8.82	9.82	0.45
Elementary occupations	10.63	11.39	4.29
Total	100.00	100.00	100.00
Number of obs	64,970	55,776	9,194

Source: authors’ calculation.

### 4.1. Mismatch incidence

Table 2 shows the presence of both over and under-education in the labor market. However, the under-education is a more noticeable mismatch. Around 45.34% of men in Pakistan are in jobs that are mismatched to their obtained level of education, with 29.98% being undereducated and about 14.82% over-educated. Compared to male workers, statistics based on the data used in this study show that 59.74% of females are working as regularly paid employees in occupations (that require at least 35 hours of work per week) that are well-matched to their attained level of education. 40.26% of females face a job-education mismatch in the labor market, with 37.38% can be categorized as under-educated compared to the required level of education for a specific occupation. In comparison, only 2.88% of females are classified as over-educated compared to the required level of education in a particular occupation.

**Table 2:** Incidence of educational mismatch.

	Total	Male	Female
Over-educated	14.82	16.25	2.88
Matched	55.20	54.66	59.70
Under-educated	29.98	29.09	37.38
total	100.00	100.00	100.00

Source: authors’ calculation.

The distribution of mismatch across years of working experience (Table 3) shows that the rate of over-education decreases significantly with years of experience, from 27.66% to 15.43 for males and 3.18 to 2.60% for females. The under-education seems low among less experienced, while it’s significantly high among the labor force with more years of experience. These results are in accordance with the human capital theory. The years of experience can be treated as an alternate form of human capital and substitute the level of education required for a job [10, 26].

**Table 3:** Incidence of mismatch by experience.

Age	Experience	Under-education		Over-education	
		Male	Female	Male	Female
15–65	0–05	23.56	32.89	17.66	3.18
	6–10	26.43	34.61	18.65	2.64
	11–15	27.92	40.45	17.38	2.45
	16–25	29.59	45.98	14.39	2.38
	26–35	34.05	49.24	14.62	2.42
	36–45	43.67	73.27	15.43	2.60

Source: authors’ calculation.

Statistics of educational mismatch across occupational groups (Table 4) reveal the highest under-education rate among the male labor force in the ‘Clerical support workers’ category and Technicians and Associate Professionals’ among the female labor force. The highest rate of over-education among male and female labor force exists in the ‘Plant and Machine Operators and Assemblers’ category. Overall, the average incidence of educational mismatch by occupational classifications clearly shows both mismatches in the labor market. These averages don’t account for age, but they give a good idea of occupations with the more prevalent mismatch.

**Table 4:** Percentage distribution of mismatch by occupation.

Occupation	under-education		Match		Over-education	
	Male	Female	Male	Female	Male	Female
Manager	23.63	6.51	76.33	92.56	0.05	0.93
Professionals	32.80	27.75	66.96	71.97	0.24	0.27
Technicians and associate professionals	57.95	57.99	42.05	42.01	-	-
Clerical support workers	64.73	48.15	35.27	51.85.13	-	-
Service and sales workers	19.86	17.60	72.15	69.60	7.99	12.80
Skilled agricultural, forestry, and fishery workers	-	-	39.19	40.00	60.81	60.00
Craft and related trades workers	27.68	35.71	68.16	64.29	4.16	-
Plant and machine operators and assemblers	-	-	36.16	25.81	63.84	74.19
Elementary occupations	-	-	36.36	55.09	63.64	44.91

Source: authors’ calculation.

### 4.2. Return to education

First, the basic mincer model of returns to education attained by male and female workers in Pakistan is estimated. It is worth noting that, when dealing with pooled or panel nature of data, the data set may have characteristics that are shared between members of a group but differ across groups (e.g., occupational groups, industry), known as fixed effects [43, 44]. Thus, it is important to consider this unobserved heterogeneity or fixed effects. Based on the existing literature (e.g., [5, 29], this study accounts for the location of residence (if living in the rural area), province, occupation, industry, and also the years’ effect in the model. The results are presented in Table 5. Columns (1) and (2) present the estimates of the full sample with and without fixed effect. Columns (3) and (4) present the base model estimates separately for male and female workers, and columns 5 and 6 present the estimates with control fixed effects for both genders individually. Robust standard errors clustered by industry account for unobserved heterogeneity in the model. Results presented in columns (1) and (2) show the positive effect of education on income for the pooled sample. Columns (3) and (4) of Table 5 show that the return to each additional year of education is higher for that male workers than what the women workers earn for each additional year of schooling; the male workers earn around 0.90% more than females. Columns (5 and (6) show that with the inclusion of control fixed effects such as occupational and province, this earning difference increases to 1.50%. This result could be because of the difference in opportunities and preferences of both genders. The variables of marital status show that married women earn less as compared to men. Similarly, the presence of one or more dependent child at home negatively affects the earnings of women, while male workers seem to earn more; this shows the dominant gender roles in the society where man is assumed to be primary breadwinners and have to support the family while women are considered being the secondary earner in the family.

**Table 5:** Return to education by the attained level education.

	1	2	3	4	5	6
	Full sample	Full sample	Female	Male	Female	Male
years of education	0.027*** (0.022)	0.014*** (0.0018)	0.05*** (0.005)	0.058*** (0.002)	0.036*** (0.0031)	0.051*** (0.003)
exp	0.053*** (0.005)	0.041*** (0.009)	0.023*** (0.009)	0.028*** (0.009)	0.016** (0.009)	0.024*** (0.008)
exp^2^	-0.008*** (0.0001)	-0.00006** (0.0001)	-0.0005*** (0.00008)	-0.0006*** (0.0001)	-0.0004 *** (0.00006)	-0.0004*** (0.00008)
married	-	0.027*** (0.013)	-	-	-0.02*** (-0.005)	0.04*** (.001)
child	-	-0.23*** (0.10)	-	-	-0.03*** (0.005)	0.09*** (0.003)
Residing in a Rural area	n	y	n	n	y	y
Province	n	y	n	n	y	y
Occupation	n	y	n	n	y	y
Industry	n	y	n	n	y	y
year	n	y	n	n	y	y
constant	1.66 (0.13)	1.89 (0.199)	1.98 (.009)	1.76 (.012)	1.50 (.009)	1.87 (.012)
R^2^	0.41	0.60	0.34	0.35	0.45	0.47
Prob > F	0	0	0	0	0	0
No of observation	64,964	64,964	9,192	55,772	9,192	55,772

Note: (Robust Standard Error clustered by the industry are given in parentheses).

*p < 0.05

**p < 0.01

***p < 0.001.

### 4.3. Impact of educational mismatch on earning

This section presents the estimates of ORU specification based on Eq (4). Table 6 reports the results. The robust standard error clustered by industry is used to avid across industry correlation and heteroskedasticity. Throughout this study, ORU specifications based on the mode method are used for the estimations.

**Table 6:** Return to under, required, and over education.

	1	2	3	4	5	6
	Full sample	Full sample	Female	Male	Female	Male
Matched	0.09*** (0.007)	0.05*** (0.006)	0.077*** (0.0014)	0.081*** (0.005)	0.071*** (0.0023)	0.085*** (0.0011)
Over-education	0.05** (0.01)	0.033**(0.003)	0.039*** (0.009)	0.039*** (0.005)	0.026*** (0.001)	0.032 (0.0018)
Under-education	0.06*** (0.018)	0.024*** (0.010)	0.015*** (0.004)	0.024*** (0.014)	0.011*** (0.02)	0.018** (0.002)
exp	0.038*** (0.005)	0.035** (0.008)	0.044*** (0.004)	0.053*** (0.003)	0.041*** (0.003)	0.048*** (0.004)
exp^2^	-0.0006* (0.0001)	-0.0005** (0.0001)	-0.0003*** (0.00007)	-0.0005*** (0.00007)	-0.0003*** 0.00006)	-0.0003*** (0.00005)
child	0.043** (0.008)	-0.030** (0.011)	-0.034*** (0.005)	0.057*** (0.003)	-0.031*** (0.004)	0.81*** (0.002)
Married	0.012** (0.006)	0.07** (0.01)	-0.05*** (0.02)	0.026*** (0.05)	-0.087*** (0.01)	0.023* (0.05)
Living in a Rural area	n	y	n	n	y	y
Province	n	y	n	n	y	y
Occupation	n	y	n	n	y	y
Industry	n	y	n	n	y	y
year	n	y	n	n	y	y
constant	1.12 (0.06)	2.13 (0.08)	1.10 (0.098)	1.12 (0.016)	1.97 (0.053)	1.04 (0.010)
R^2^	0.27	0.57	0.32	0.35	0.41	0.43
Prob > F	0	0	0	0	0	0
No of observation	64,964	64,964	9,192	55,772	9,192	55,772
H1	33.69***	9.77***	25.68***	43.45***	12.87***	32.47***
H2	11.76***	55.44***	26.75***	11.89***	40.38***	48.00***
H3	57.67***	72.94***	132.62***	490***	39.59***	109***

Note: (Robust Standard Error clustered by the industry are given in parentheses).

*p < 0.05

**p < 0.01

***p < 0.001.

Table 6 includes estimates of models with and without control fixed effect. Colum 1 and 2 present the results of the base explanatory model for a pooled sample before splitting it by gender, including years of experience, square terms of experience, dummy variable if married, and a dummy of dependent children at home. Columns (3) and (4) present the estimates based on ORU specification without control fixed effects, while columns (5) and (6) present the results that include control fixed effects. Columns (5) and (6) show that the wage penalty of being over-educated is higher for females (1.9%) as compared to males (2.8%); the over-educated females earn 0.9% less than the over-educated male workers. The results also show the wage premium of under-education. However, the wage premium of under-education is significantly low compared to those who have the required level of education for both male and female workers. Gender comparison shows that the wage premium for females is lower than for males. The positive return to under-education shows the skill shortage in the labor market of Pakistan, and this could be one of the major reasons for low labor productivity in Pakistan.

ORU specification provides better estimates of the return to education than the standard mincerian earnings equation (Table 5). Column (6) in Table 6 shows that Years of required level show higher return (8.5%) for male workers as compared to (5.1%) in Table 5 column (6). Similarly, column (5) in Table 6 shows a higher rate of return for females with the matched level of education compared to the estimates of standard mincerian wage function (column 5, Table 5). From the comparison of estimates provided in Tables 5 and 6, it is evident that the conventional mincerian income function is monotonic, i.e., it takes the relationship between education and earnings as linear and strictly increasing. Whereas, in the augmented mincerian model based on ORU specification, the relationship between education and earning is not monotonic because over and under-education result in lower returns than the years of education required for a specific job. This comparison of ORU specifications and mincerian wage function is in line with [5, 15, 45, 46] for the USA, Australia, UK, and New Zealand, respectively.

An attractive feature of this ORU specification is that it fits in the Human capital, job competition, or assignment model. It allows testing for three different hypotheses by applying different restrictions as:

$$H1:\;\beta_{1}=\beta_{2}=‐\beta_{3}$$ (5)

$$H2:\;\beta_{1}=\beta_{3}=0$$ (6)

$$H3:\;\beta_{1}=\beta 2=\beta 3=0$$ (7)

Eq (5) implies that return to over, under, and required level of education is equal in determining the earning. Failure to reject the null hypotheses supports the evidence of the human capital model. Eq (6) is consistent with the job competition model, and failure to reject the null hypothesis implies that earnings are only determined by the required level of over-education. The third hypothesis in Eq (7) means that the job characteristics and the extent of human capital investment jointly determine the worker’s earnings. The joint f-statistics and p values for all three hypotheses are presented in Table 6. H1: that human capital model held in the labor market of Pakistan is rejected by sample data.

Similarly, H2 and H3 are also rejected, indicating that the job market of Pakistan can be explained by the assignment model. These findings are in line with numerous previous studies, such as [5, 17, 42, 46–48]. According to the assignment model, over-education (under-education) is caused due to allocation of workers into jobs that are not well suited to their attained qualifications [14]. As it is evident that mismatch is high in Pakistan, it suggests that lower returns to over-and under-education can be attributed to inefficient job allocation in the labor market. Finally, the assignment model asserts that workers choose jobs to maximize their income (and/or utility). This notion may also explain the high rate of overseas migration by Pakistani workers who might choose to work in a foreign country instead of their home country.

The analysis presented in this study aims to identify the relationship between earnings and each year of over, required, or under-education. It is important to acknowledge that several unobservable individual characteristics can endogenously influence the returns to over, required, or under-education. However, because of data limitations, the analysis presented in this study cannot distinguish the extent to which the unobservable factors, such as unobserved ability, productivity, and other skills relevant for the labor market, can influence the returns to over, required, or under-education. Nevertheless, using the standard modeling approach, this study presents the extent of educational mismatch in the labor market and the potential earnings differentials associated with it.

Given the Population structure of Pakistan, an important aspect worth further consideration is the effect of educational mismatch on the income of various age groups. Table 7 presents the estimates of the return to education based on ORU specification by different age groups. The Table shows that the returns to over, required, and under-education are not sensitive to generational changes. The estimates presented in Table 7 is consistent with the main findings presented in Table 6.

**Table 7:** Return to under, required, and over-education by age.

ORU	15–25		25–45		45–65	
	Female	Male	Female	Male	Female	Male
Over	0.024*** (0.0013)	0.040*** (0.0025)	0.023*** (0.009)	0.030*** (0.0019)	0.013*** (0.0061)	0.015*** (0.0037)
Required	0.058*** (0.0014)	0.049*** (0.002)	0.048*** (0.008)	0.046*** (0.00295)	0.050*** (0.0015)	0.030*** 0.0034)
Under	0.037*** (0.002)	0.042** (0.003)	0.042*** (0.003)	0.036* (0.003)	0.042*** (0.006)	0.039** (0.005)
R^2^	0.32	0.37	0.35	0.37	0.38	0.39
Prob > F	0	0	0	0	0	0

Note: (1) Robust Standard Error clustered by the industry are given in parentheses. (2) This Table only reports the estimates of ORU specifications. The model estimation includes the complete set of variables and a constant term as in Table 5.

*p < 0.05

**p < 0.01

***p < 0.001.

## 5. Conclusion

The educational mismatch has become one of the major challenges in labor market policy in recent years. This paper investigates the effect of educational mismatch on earnings in the labor market of Pakistan. Based on the Labor force survey of Pakistan, the results provide an insight into educational mismatch and its effect on earning distribution for the employed labor force across genders and occupations. The findings of this study show that both over and under-education persist in the labor market of Pakistan. These findings are similar to previous literature dealing with developing countries such as Vietnam [29] and the Brazilian labor market [37]. The earning penalty of under-education is more significant as compared to over-education. Even though years of experience compensate for education mismatch, it is not the perfect substitute for the required level of education. The results also indict the labor market of Pakistan functions according to the assignment theory model.

Even though this study is limited to vertical educational mismatch, it has important implications for the labor market of Pakistan as it provides detailed information about the incidence of vertical educational mismatch and the earning penalties associated with that mismatch.

The findings of the study lead to the following policy implications and recommendations:

The incidence of educational mismatch highlights that: (1) the education system is not coping with the right demands of the labor market, and; (2) the labor force also lacks accurate information about the job market. It is imperative to introduce an organized information system that provides more accurate information about the employment patterns, skills, and education in demand in the labor market and improves the matching process. For the female labor force, it is also important to address the socio-cultural barriers and labor market discrimination. There is also a need to raise incentives to improve the job search intensity. And to overcome the negative effect of under-education, upskilling through on-the-job training to increase productivity is of utmost importance to raise the earnings level of workers.

## Supporting information

S1 Appendix (DOCX)
