# Supplementary material for: Incidence of educational mismatch and earning in Pakistan
Source: PLoS One. 2022 Jun 3;17(6):e0268008. doi: 10.1371/journal.pone.0268008 (PMC9165871; doi:10.1371/journal.pone.0268008)
Supplement: S1 Appendix — (DOCX) [file pone.0268008.s001.docx]

**Appendix**

| **Table A1: List of Variables** | |
| --- | --- |
| **Label** | **Detail** |
| Log m_i | Log of monthly income from salary/ wage |
| Years of education | Attained level of education an individual |
| Required | Years of education required for an occupation |
| Over | Years of obtained education exceeds the years of required-education for an occupation |
| Under | For an occupation, years of education are required. |
| exp | Years of experience |
| exp^2^ | Square term of years of experience |
| child | Number of dependent children |
| married | dummy variable = 1 if married, 0 otherwise |
| Living in Rural area | dummy variable = 1 if residing in Urban area, 0 otherwise |
| Province dummies | The four provinces include Sindh, Balochistan, KPK, and Punjab |
| Occupation dummies | 9 major occupations include Manager, Professionals, Technicians and associate professionals, Clerical support workers, Service and sales workers, Skilled agricultural, forestry, and fishery workers, Craft and related trades workers, Plant and machine operators, and assemblers and Elementary Occupations. |
| Industry dummies | 17 industries include Agriculture, hunting, and forestry, fishing, Mining and quarrying, Manufacturing, electricity, gas, and water supply, Construction, Wholesale and retail trade, Hotels and restaurants, Transport and storage, Communications, Financial intermediation, real estate, renting, and business activities, Public administration and defense, Education, Health and social work, other community, social and personal service activities, and all other (uncategorized). |
